# Dynamic Response Study of Piezoresistive Ti_3_C_2_-MXene Sensor for Structural Impacts

**DOI:** 10.3390/s23208463

**Published:** 2023-10-14

**Authors:** Shreyas Srivatsa, Paul Sieber, Céline Hofer, André Robert, Siddhesh Raorane, Marianna Marciszko-Wiąckowska, Krzysztof Grabowski, M. M. Nayak, Eleni Chatzi, Tadeusz Uhl

**Affiliations:** 1 Space Technology Centre, AGH University of Science and Technology, 30-059 Krakow, Poland; 2 Academic Centre for Materials and Nanotechnology, AGH University of Science and Technology, 30-059 Krakow, Poland; 3 Department of Civil, Environmental and Geomatic Engineering, ETH Zurich, 8092 Zurich, Switzerland; 4 Department of Robotics and Mechatronics, AGH University of Science and Technology, 30-059 Krakow, Poland; 5 Centre for Nano Science and Engineering, Indian Institute of Science, Bengaluru 560012, India

**Keywords:** 2D nanomaterials, MXenes, impact sensors, piezoresistive, structural health monitoring

## Abstract

MXenes are a new family of two-dimensional (2D) nanomaterials. They are inorganic compounds of metal carbides/nitrides/carbonitrides. Titanium carbide MXene (Ti${}_{3}$C${}_{2}$-MXene) was the first 2D nanomaterial reported in the MXene family in 2011. Owing to the good physical properties of Ti${}_{3}$C${}_{2}$-MXenes (e.g., conductivity, hydrophilicity, film-forming ability, elasticity) various applications in wearable sensors, energy harvesters, supercapacitors, electronic devices, etc., have been demonstrated. This paper presents the development of a piezoresistive Ti${}_{3}$C${}_{2}$-MXene sensor followed by experimental investigations of its dynamic response behavior when subjected to structural impacts. For the experimental investigations, an inclined ball impact test setup is constructed. Stainless steel balls of different masses and radii are used to apply repeatable impacts on a vertical cantilever plate. The Ti${}_{3}$C${}_{2}$-MXene sensor is attached to this cantilever plate along with a commercial piezoceramic sensor, and their responses for the structural impacts are compared. It is observed from the experiments that the average response times of the Ti${}_{3}$C${}_{2}$-MXene sensor and piezoceramic sensor are 1.28±0.24 μs and 31.19±24.61 μs, respectively. The fast response time of the Ti${}_{3}$C${}_{2}$-MXene sensor makes it a promising candidate for monitoring structural impacts.

## 1. Introduction

Structural health monitoring (SHM) [1] forms a critical part of engineering structures facilitating/providing enhanced life-cycle operation with cost-effectiveness. SHM requires the deployment of various sensors to measure the field variables (such as displacement, strain, pressure, temperatures, etc.) experienced by the structures. Among these various sensors, an impact sensor detects the impact as quickly as possible to indicate an event affecting the operation of the structure. An impact is a short-duration (likely in nano or micro or milli seconds) force acting on a structure [2]. For example, impacts such as bird hits on aerospace structures, automobile crash incidents, touchdown of aircraft landing gear on the ground, space debris collision with satellites, etc., pose a danger to the normal operation of structures. Monitoring such impact events requires sensors that have dynamic response properties capable of capturing the event without loss of information. Among the dynamic response properties, response time plays a significant role in impact event monitoring, as the response of the sensor needs to be fast enough to trigger a control activity (e.g., airbag deployment due to an automobile crash or control system readjustment owing to aerospace structural damage). Several impact sensors have been developed in the literature [3]. In this paper, we investigate the dynamic response behavior of MXene nanomaterial-based sensors in monitoring structural impacts.

With the reporting of many nanomaterials over the last few decades, including carbon-based nanomaterials [4], boron nitride, molybdenum sulfide, etc. [5], the use of these multifunctional nanomaterials as sensing elements provides the advantages of compactness, structure surface conformability and high sensitivity for applications in SHM. Among these nanomaterials, MXenes are the latest family of nanomaterials being proposed for SHM applications [6]. Titanium carbide MXene (Ti${}_{3}$C${}_{2}$-MXene) was the first reported inorganic compound in the MXene family in 2011 [7]. Since then Ti${}_{3}$C${}_{2}$-MXene has been used in sensor development for human wearable sensors [8] and biosensors [9]. Recently, the dynamic response properties of pure Ti${}_{3}$C${}_{2}$-MXene film were reported and observed to be as good as piezoceramic commercial sensors [10]. The use of Ti${}_{3}$C${}_{2}$-MXene as sensors in SHM for measuring strain, temperature, and pressure are thoroughly discussed in [6]. The authors in [6] also provide a clear connection between MXene nanomaterial properties and their use as sensors. To investigate the possibility of using Ti${}_{3}$C${}_{2}$-MXene as dynamic sensors for SHM, in this paper, an impact sensor is developed and tested with a new table-top impact experimental setup.

The table-top impact experimental setup constructed for the purpose of testing the impact sensors is portable and cost-effective. In this experimental setup, a stainless steel ball rolls down an inclined rail and impacts a vertically mounted cantilever aluminum plate. In this paper, stainless steel balls of different masses and radii are employed, and the impact events owing to the balls are measured using Ti${}_{3}$C${}_{2}$-MXene and piezoceramic commercial sensors attached to the cantilever plate. The responses of the Ti${}_{3}$C${}_{2}$-MXene and piezoceramic sensors are analyzed to obtain their respective response times and peak response voltages with respect to different (mass and radius) ball impacts.

The organization of this paper is described henceforth. First, a brief description of the Ti${}_{3}$C${}_{2}$-MXene preparation and sensor fabrication method is reported followed by the description of the experimental setup. The experimental theory concerning the inclined ball impact on the vertical cantilever plate is provided. Next, the material characterization of Ti${}_{3}$C${}_{2}$-MXene is reported to confirm the successful synthesis of the nanomaterial. Subsequently, the response times and sensitivities of Ti${}_{3}$C${}_{2}$-MXene and commercial piezoceramic sensors are reported and compared. Finally, the advantages and limitations of the Ti${}_{3}$C${}_{2}$-MXene sensor for applications in SHM are discussed and the conclusions are reported.

## 2. Materials and Methods

### 2.1. $Ti_{3}C_{2}$-MXene Preparation

Ti${}_{3}$C${}_{2}$-MXene was synthesized using a water-based and acid-based chemical etching process. In-situ formation of hydrogen fluoride [11] formed due to a mixture of hydrogen chloride (HCl) and lithium fluoride (LiF) was used in etching titanium aluminum carbide (Ti${}_{3}$C${}_{2}$-MAX phase compound, <40 microns particle size, Materials Research Center, Kyiv, Ukraine). The etchant solution was formed with the addition of 9 M HCl (Honeywell Fluka, Charlotte, North Carolina), 5 mL deionized water (Chempur, Karlsruhe, Germany), and 1.5 g of LiF (Sigma-Aldrich, St. Louis, MI, USA). A total of 1 g of Ti${}_{3}$C${}_{2}$-MAX compound was added slowly over 5–10 min into the etchant liquid, which was stirred at 400 RPM at 35 °C for 24 h [12]. Once the reaction was completed, the solution was washed with deionized water and centrifugated at a speed of 4500 RPM to precipitate the multilayer Ti${}_{3}$C${}_{2}$-MXene, and the clear supernatant was decanted. The washing cycle was continued till the pH of the supernatant reached a neutral value. Further, deionized water was added to the thick paste of multilayer MXene to employ a mildly intensive layer delamination (MILD) method to vigorously shake the colloidal solution for 10 mins to finally obtain the delaminated Ti${}_{3}$C${}_{2}$-MXene colloidal solution.

### 2.2. Impact Sensor Fabrication

**Figure 1:**
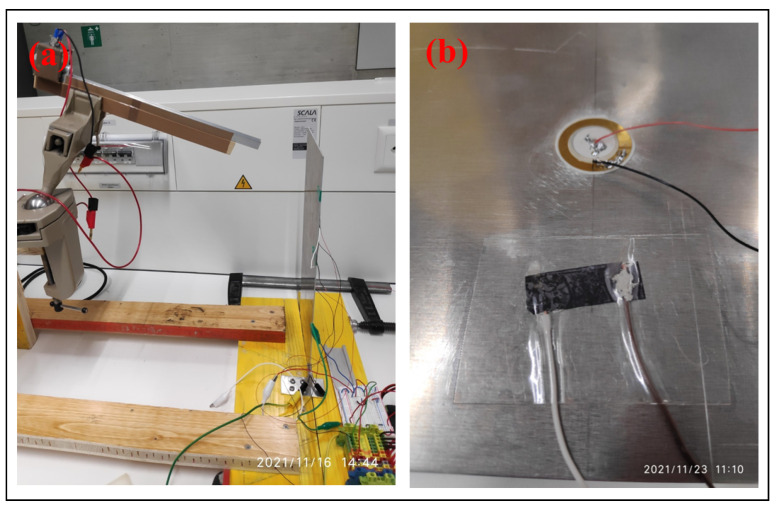
(**a**) Photograph of the experimental test setup. (**b**) Ti${}_{3}$C${}_{2}$-MXene and piezoceramic sensors attached to the plate.

The Ti${}_{3}$C${}_{2}$-MXene colloidal solution obtained by the preparation method discussed above was then passed through a nylon filter paper (GE Healthcare, Whatman, Kent, United Kingdom) in a vacuum-assisted filtration setup. The delaminated Ti${}_{3}$C${}_{2}$-MXene nanosheets self-assemble under the influence of differential pressure to form a film of Ti${}_{3}$C${}_{2}$-MXene. The films produced were vacuum-dried at 60 °C for 24 h. These films were then cut into rectangular shape samples (14×9 mm; 14 μm thickness). Silver epoxy paste (Sigma-Aldrich, St. Louis, MO, USA) was used to connect the wires for making the Ti${}_{3}$C${}_{2}$-MXene films into a two-terminal sensor. This assembly was placed between polyethylene terephthalate (PET) films (110 microns thick) and laminated to form an impact sensor with Ti${}_{3}$C${}_{2}$-MXene sensing element (similar to [10]). The Ti${}_{3}$C${}_{2}$-MXene impact sensor fabricated had a resistance value of 220 ± 0.5 Ω on the day of the experiment (23 November 2021). The Ti${}_{3}$C${}_{2}$-MXene sensor was connected to a Wheatstone bridge configuration with three other 220 Ω resistors to convert the resistance change to voltage change, similar to [10]. Figure 1b shows the fabricated impact sensor attached alongside the commercial piezoceramic (lead zirconate titanate or PZT, 14 mm diameter) sensor. The output signals obtained without amplification from these sensors were connected to the Digilent Analog Discovery 2 data acquisition system to record the data. Further processing of data was performed through Matlab 2022b and Origin 2022b software to obtain the sensor characteristics reported in Section 3.2.

### 2.3. Experimental Structural Impact Setup

**Figure 2:**
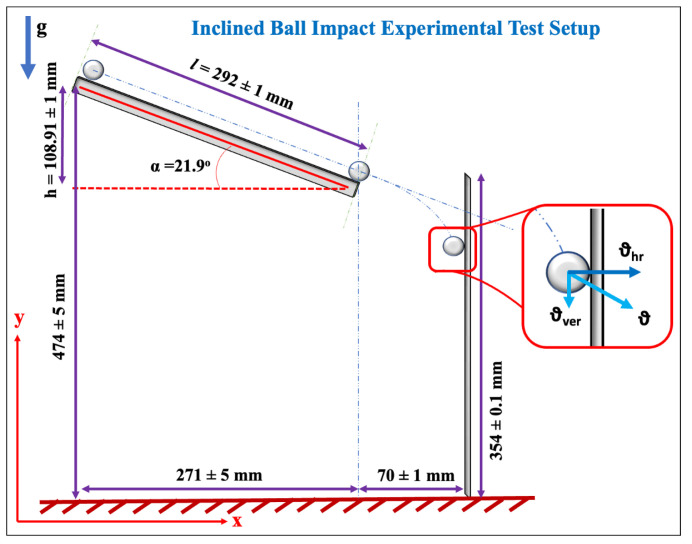
Illustration of the table-top experimental structural impact test setup.

A table-top experimental setup was constructed for the application of repeatable impact force on a structure (shown in Figure 1a and Figure 2). The wooden board-based support structure (thickness of the wooden boards: 26 mm) was constructed for the advantage they offer due to portability and affordability. The vertical cantilever aluminum plate (total plate dimensions: 380×160×1.49 mm; mass of plate: 246.521 g) was fixed using wooden boards (length of the plate constrained: 26 mm) and alignment clamps, as shown in Figure 1a. The fixing of the vertical plate results in constraining the displacement and rotation of one end of the plate, creating a fixed essential (or geometric) boundary condition (effective length of the plate after fixing: 354±0.1 mm) while the other end of the plate was free to vibrate. Ti${}_{3}$C${}_{2}$-MXene and piezoceramic sensors were attached to the plate on the opposite side that was subjected to the ball impacts. The placement of sensors (at the location measured from the top free edge of the plate: 100×80 mm) was neither close to the free-end or fixed-end to avoid the end effects (according to Saint Venant’s principle [13]).

The stainless-steel balls were placed at the top of the inclined rail (length of the rail, *l*, is 292±1 mm) (using an electromagnet) to allow the ball to roll down to impact the vertical cantilever plate (as shown in Figure 1a and Figure 2). To apply a single impact onto the vertical cantilever plate, the inclined ball setup was constructed to allow the ball to impact and rebound off the plate. A total of six balls with different radii and masses (listed in Table 1) were used in this experimental work. The subsequent section discusses the experimental theory to calculate the impact force acting on the vertical cantilever plate.

### 2.4. Brief Experimental Theory

In order to employ impact sensors for accurate measurements, it is crucial to calibrate them. For calibration, the impact force exerted by the ball on the plate needs to be determined. For the development of an initial mathematical model, the following assumptions are made about the experiment setup:

- Dynamics of the ball on the rails: No-slip and no-bounce conditions of the ball along with negligible rolling resistance when the ball rolls down the ramp.
- Ball-Plate interaction: Collision between the ball and the cantilever plate is assumed to be an elastic collision neglecting all thermal effects due to the collision.
- Projectile motion of the ball: The trajectory of the ball in the space between the rail and the plate follows a parabolic path in the x-y plane (shown in Figure 2). (Assuming the absence of air resistance.)
- Linear mechanics assumptions: The impact is assumed to generate a small strain and small deformation response of the plate structure.
- Dynamics of the plate: Upon impact from the ball, the plate is assumed to have only transverse vibration in the x-y plane—resulting in a cantilever-type response of the vertical plate.

Considering the two-dimensional mechanics of the complete system, the mathematical theory is developed to estimate the average impact force that acts on the plate. In this paper, the theoretical development focuses only on the impact event while the consideration of other aspects of the mechanics of the problem is reserved for future works. Upon jumping off the end of the rail, the vertical velocity component of the ball changes due to acceleration due to gravity while the horizontal velocity component remains the same [14]. It is assumed that only the horizontal velocity component of the ball causes the transverse deflections of the vertical cantilever plate.

For calculating the impact force, there is a need to determine the impact velocity followed by the impact time. Assuming no loss of energy, the potential and kinetic energies relate as

(1) $$mgh=\underset{T}{\underbrace{\frac{mv^{2}}{2}}}+\underset{R}{\underbrace{\frac{\omega^{2}mr^{2}}{5}}},$$

where the left-hand side and right-hand side terms represent potential energy and kinetic energy, respectively, and the terms with underbrace *T* and *R* represent translational and rotational kinetic energies, respectively; *m* denotes the mass of the ball, *g* denotes the acceleration due to gravity, *h* is the height of the ramp (please see Figure 2), and ω and *v* are the angular and translational velocities, respectively. Then, using the relation of v=rω, the ball’s impact velocity can be obtained as

(2) $$v=\sqrt{\frac{10gh}{7}}.$$

Next, to determine the impact time, it was assumed that the horizontal component of *v* becomes 0 at impact. Then, using the equation available in [2], the impact time of the ball, $t_{0}$, is obtained as

(3) $$t_{0}=2.94{\left(\frac{5}{4Mn\sqrt{v_{hr}}}\right)}^{2/5},$$

where $M=1/m_{1}+1/m_{2}$ represents the total mass with $m_{1}$ and $m_{2}$ denoting the masses of the ball and the plate, respectively, and $v_{hr}=v\cos\left(\alpha \right)$ represents the horizontal component of the impact velocity. Furthermore,

(4) $$n=\frac{4\sqrt{r}}{3\pi (k_{1}+k_{2})},$$

where *r* is the radius of the ball, and $k_{i}=\frac{1-\nu_{i}^{2}}{\pi E_{i}}$ with i∈{1,2}; *E* and ν represent Young’s modulus and Poisson’s ratio, respectively, and the subscripts 1 and 2 represent the ball and plate, respectively.

Relating momentum with impulse, the impact force is determined as (note the assumption of the horizontal component of impact velocity becoming 0 at impact)

(5) $$F=\frac{mv_{hr}}{t_{0}},$$

where *F* represents the average impact force. Using the above formulas, the results presented in Table 1 were obtained. For different balls impacting the plate, it can be observed that, as the size of the ball decreases, i.e., the radius and the mass of the ball decreases, the impact time and the impact force decrease. Note that the impact velocity remains the same for all the balls (please see Equation (2)) such that v=1.2360 m/s.

## 3. Results and Discussions

### 3.1. X-ray Diffraction

**Figure 3:**
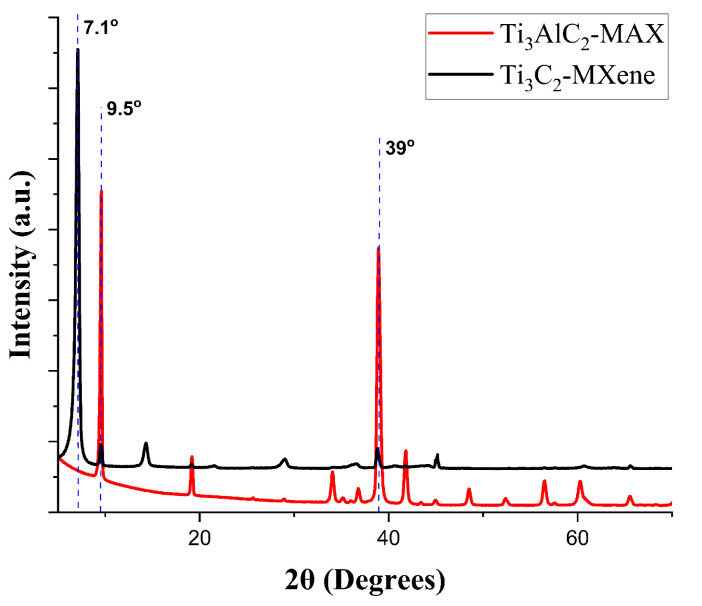
XRD analysis of Ti${}_{3}$C${}_{2}$-MAX and Ti${}_{3}$C${}_{2}$-MXene film for post-synthesis confirmation.

X-ray diffraction (XRD) is a critical study for confirming the production of Ti${}_{3}$C${}_{2}$-MXene [12]. The morphological studies using a scanning and transmission electron microscope for the Ti${}_{3}$C${}_{2}$-MXene produced by the authors can be found in [6,10,15]. The XRD analyses of the Ti${}_{3}$C${}_{2}$-MAX and Ti${}_{3}$C${}_{2}$-MXene (shown in Figure 3) are performed to confirm the complete etching of aluminum from Ti${}_{3}$C${}_{2}$-MAX to form 2D MXenes. With the removal of aluminum, the peaks of Ti${}_{3}$C${}_{2}$-MXene broaden, and the characteristic peak of aluminum close to 39° disappears in the Ti${}_{3}$C${}_{2}$-MXene film sample. In Figure 3, the peak at (002) shifts from 9.5° to 7.1°. The d-spacing change also indicates the intercalation of layers of water molecules between the delaminated Ti${}_{3}$C${}_{2}$-MXene nanosheets. This confirms the successful synthesis of Ti${}_{3}$C${}_{2}$-MXene [10,12].

### 3.2. Response and Comparison of Sensors

**Figure 4:**
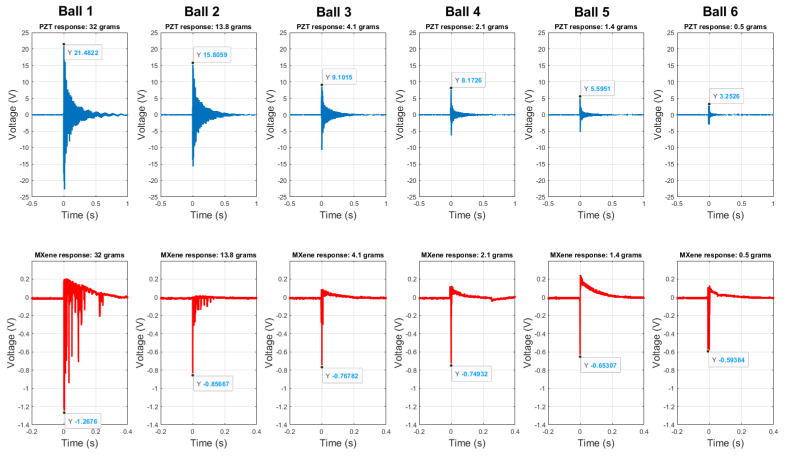
Comparison of the response of Ti${}_{3}$C${}_{2}$-MXene and piezoceramic sensor raw data for the balls 1 to 6 (single trial plotted) listed in Table 1.

Experiments are performed multiple times with each ball to impact the plate and obtain the response of the sensors. The responses of Ti${}_{3}$C${}_{2}$-MXene and piezoceramic sensors for each ball impact are shown in Figure 4 (For the sake of brevity, a single trial for each ball impact has been shown in the figure here). It can be observed from Figure 4 and Table 1 that with the decrease in the mass of the ball impacting the plate (from Ball ID 1 to Ball ID 6), there is a decrease in impact force and impact time resulting in a decrease in the peak response voltage of the sensors.

#### 3.2.1. Sensitivity

**Figure 5:**
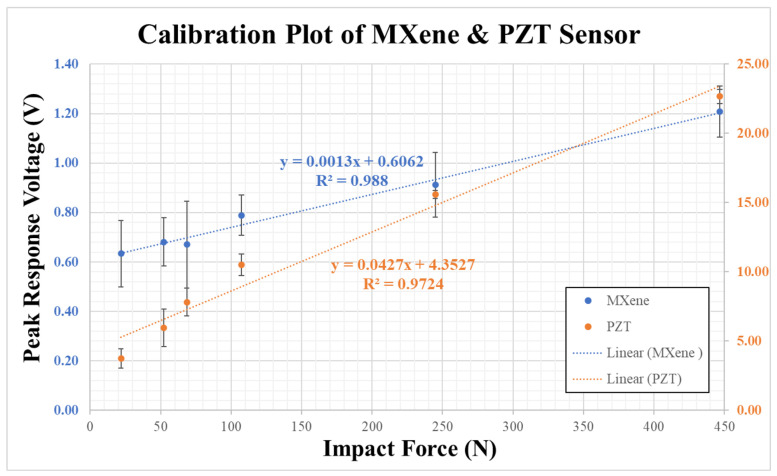
Peak Response Voltage plotted against impact force to determine the calibration curve and sensitivity of Ti${}_{3}$C${}_{2}$-MXene and piezoceramic sensors.

The response signals and data from the sensors are post-processed with Matlab 2022b and Origin software 2022b to obtain the sensor response characteristics and determination of response time. The peak response voltages of Ti${}_{3}$C${}_{2}$-MXene and piezoceramic sensors (referred to as PZT) are tabulated in Table 1 with the mean and standard deviations obtained with repeatability tests. The calibration curves of Ti${}_{3}$C${}_{2}$-MXene and piezoceramic sensors with the least square fit (with $R^{2}>95\%$) are shown in Figure 5. The sensitivity of Ti${}_{3}$C${}_{2}$-MXene and piezoceramic sensors were found to be 1.3 mV/N and 42.7 mV/N, respectively. It is to be noted that the sensitivity of Ti${}_{3}$C${}_{2}$-MXene piezoresistive sensor is lower compared to the commercial piezoceramic sensor. This is due to the fact that the output signal of the piezoresistive Ti${}_{3}$C${}_{2}$-MXene sensor is not amplified. For the commercial application of this Ti${}_{3}$C${}_{2}$-MXene material, optimization of sensing element properties, as well as post-processing of sensor signal, is needed. These are beyond the scope of this paper and are reserved for future work.

#### 3.2.2. Response Time

The average response times for all the trials of Ti${}_{3}$C${}_{2}$-MXene and piezoceramic sensors are 1.28±0.24 μs and 31.19±24.61 μs, respectively. Table 1 provides the mean and standard deviations of the response time for both sensors obtained with repeatability tests. The trend of a decrease in response time of the sensors with decreasing impact force is noted in the table. However, the response time of the commercial piezoceramic sensor was an order of magnitude higher than the Ti${}_{3}$C${}_{2}$-MXene sensor. Thus, indicating the capability of the Ti${}_{3}$C${}_{2}$-MXene sensors for fast sensing of structural impacts.

### 3.3. Discussions

In this paper, the responses of Ti${}_{3}$C${}_{2}$-MXene and piezoceramic sensors are subjected to simple statistical analysis. Table 2 presents all the latest work in the literature on Ti${}_{3}$C${}_{2}$-MXene along with the results from this paper. The fast response times of Ti${}_{3}$C${}_{2}$-MXene sensor reported in this work (1.28±0.24 μs) outperform most of the reported works in the literature. The Ti${}_{3}$C${}_{2}$-MXene sensor also has comparable performance with commercially available piezoelectric sensors (3 μs) used in industry for dynamic sensing [16]. This paper is the first demonstration of the Ti${}_{3}$C${}_{2}$-MXene impact sensor and its use in structural impact monitoring.

There are several limitations of the Ti${}_{3}$C${}_{2}$-MXene impact sensor in the present form. The lower sensitivity compared to piezoceramic sensors needs to be addressed with better signal processing as well as optimization of sensor characteristics. For applications in on-field SHM systems such as crash detection or aerospace structural integrity monitoring, the long-term performance of these Ti${}_{3}$C${}_{2}$-MXene sensors needs to be studied. The effects of aging, reliability, and robustness of the sensors are some of the assessments needed for establishing Ti${}_{3}$C${}_{2}$-MXene sensors as a natural choice for SHM.

The new experimental setup, proposed in this paper, has been designed and constructed to be portable and cost-effective. However, the analytical/mathematical model developed in this work is a preliminary step, as the complete mechanics involved in the operation of the setup have not been captured. These include the dynamics of the plate after impact, contact mechanics between the ball and plate, etc. To overcome the limitations of this model, numerical methods can be employed for better estimation of the impact force, which is critical in the calibration of the sensor. Further, the mathematical model needs to be validated with experimental results to establish the experimental setup as a benchmark problem for the future. These limitations of this work will be taken up for consideration in the future. Reporting of these initial results on Ti${}_{3}$C${}_{2}$-MXene sensors for SHM provides a foreground to establish Ti${}_{3}$C${}_{2}$-MXene nanomaterial as a suitable material for next-generation sensors.

## 4. Conclusions

The paper reports the development and testing of a Ti${}_{3}$C${}_{2}$-MXene sensor. This work is the first reporting of Ti${}_{3}$C${}_{2}$-MXene sensor used for monitoring engineering structures. In this paper, a Ti${}_{3}$C${}_{2}$-MXene impact sensor is developed and tested along with a commercial piezoceramic sensor. A new table-top experimental setup is constructed to apply repeatable impact force and obtain the responses of the sensors. With a fast response time of about 1.28±0.24 μs, Ti${}_{3}$C${}_{2}$-MXene sensor demonstrated a sensitivity of 1.3 mV/N without signal amplification. The response times of Ti${}_{3}$C${}_{2}$-MXene sensor were an order of magnitude lower than the commercial piezoceramic sensor. The results from this investigation demonstrate the possibility of using Ti${}_{3}$C${}_{2}$-MXene sensors for structural impact monitoring.

## Figures and Tables

**Table 1:** Impact times and impact forces calculated for various balls used for test with response parameters (PV: Peak Voltage; RT: Response Time) (using the formulas presented in Section 2.4).

Ball ID	Mass (kg)	Radius (m)	$t_{0}$ (μs)	*F* (N)	MXene PV (V)	PZT PV (V)	MXene RT (μs)	PZT RT (μs)
1	0.0326	0.01	83.70	446.62	1.20±0.10	22.67±0.51	1.70±0.40	81.33±0.40
2	0.0138	0.0075	64.64	244.81	0.91±0.13	15.58±0.29	1.52±0.19	40.62±1.03
3	0.0041	0.005	43.80	107.34	0.78±0.08	10.52±0.77	1.26±0.62	25.59±1.18
4	0.0021	0.004	35.16	68.49	0.67±0.17	7.81±1.00	1.10±0.26	17.35±0.40
5	0.0014	0.0035	30.74	52.22	0.68±0.09	5.96±1.36	1.57±0.11	12.82±3.38
6	0.0005	0.0025	21.81	26.28	0.63±0.13	3.73±0.69	1.03±0.25	9.44±2.77

**Table 2:** Comparison of the response time of Ti${}_{3}$C${}_{2}$-MXene sensor developed in this paper with the literature.

Material	Experiment Type	Response Time
**Pure Ti${}_{3}$C${}_{2}$-MXene film**	**Inclined ball impact (Piezoresistive)**	**1.28 ± 0.24 μs (This work)**
**PZT (Commercial sensor)**	**Inclined ball impact (Piezoelectric)**	**31.19 ± 24.61 μs (This work)**
Pure Ti${}_{3}$C${}_{2}$-MXene film [10]	Shock tube test (Piezoresistive)	7.13 ± 1.28 μs
Pure Ti${}_{3}$C${}_{2}$-MXene film [10]	Ball drop test (Piezoresistive)	1.56 ± 0.03 ms
Pure Ti${}_{3}$C${}_{2}$-MXene film [17]	Compression test (Piezoresistive)	30 ms
Ti${}_{3}$C${}_{2}$-MXene/Sponge network [18]	Compression test (Piezoresistive)	130 ms
Pure Ti${}_{3}$C${}_{2}$-MXene [19]	Tensile test (Piezoresistive)	88 ms
Ti${}_{3}$C${}_{2}$-MXene nanoparticle-nanosheet hybrid [20]	Tensile test (Piezoresistive)	130 ms
Ti${}_{3}$C${}_{2}$-MXene/Polyvinyl butyral [21]	Pressure test (Piezoresistive)	110 ms
Ti${}_{3}$C${}_{2}$-MXene/PVA/Polyvinyl pyrrolidone [22]	Tensile test (Piezoresistive)	33.5 ms
Ceramic [16] (Commercial sensor)	Shock tube test (Piezoelectric)	≤3 μs
Silicon [23] (Commercial sensor)	Shock tube test (Piezoresistive)	1 ms

## Data Availability

Currently, the data presented in this study are available on request from the corresponding author. This data will be further made open access on a funding agency-approved repository.
